# *Ex vivo* mucosal cultures: an emerging player in vaccine development

**DOI:** 10.3389/fcimb.2025.1739617

**Published:** 2026-01-07

**Authors:** Nikita Chilakamarri, Erwan Sallard, Malik Aydin

**Affiliations:** 1 Laboratory of Translational Medicine and Pediatric Infectious Diseases, Center for Biomedical Education and Research (ZBAF), Department of Human Medicine, Faculty of Health, Witten/Herdecke University, Witten, Germany; 2 Virology and Microbiology, Center for Biomedical Education and Research (ZBAF), Department of Human Medicine, Witten/Herdecke University, Faculty of Health, Witten, Germany; 3 Nuffield Department of Medicine, The Jenner Institute, University of Oxford, Oxford, United Kingdom; 4 Chair of Pediatrics, Children’s Hospital, Vestische Kinder- und Jugendklinik Datteln, Witten/Herdecke University, Datteln, Germany; 5 Institute for Medical Laboratory Diagnostics, Center for Clinical and Translational Research, Helios University Hospital Wuppertal, Witten/Herdecke University, Wuppertal, Germany

**Keywords:** air-liquid-interface, *ex vivo* models, microbiota, mucosa-associated lymphoid tissue, mucosal vaccines, organoids, organ-on-chip

## Abstract

Increased awareness of pathogens with pandemic potential, especially respiratory viruses, is driving research on next-generation mucosal vaccines. However, clinical translation is still hampered by the lack of relevant experimental systems. Here, we review advances in human mucosal *ex vivo* cultures and their eligibility for vaccine development as an alternative to animal models. Ranging from organotypic air-liquid interface cultures to lymphoid organoids and microfluidics-based co-cultures, several breakthroughs occurred in recent years in modeling mucosa architecture and physiology, as well as adaptive immune responses. Advancing recent progress for clinical developments may require high-throughput approaches to validate the representativeness of the immune response within models, benchmark best practices for regulatory standardization, and investigate the influence of microbiota on mucosal immune responses.

## 1 Introduction

Infections occurring at mucosal tissues are one of the major causes of global mortality and morbidity. For example, respiratory viral infections account for approximately 4 million deaths annually, primarily in immunocompromised individuals, children, and elderly patients (Khales et al., 2025). Severe outbreaks of influenza, respiratory syncytial virus (RSV) and SARS-CoV-2 occurred simultaneously several times in the last six years, with increased healthcare burden on all continents. Influenza virus and RSV are responsible for respectively 100, 000 and 290, 000-650, 000 deaths every year (WHO.int) (B27, WHO, n.d.), while SARS-CoV-2 has led to 600 million reported cases and close to 7 million deaths from its initial outbreak (Healthdata.org). In addition to directly compromising the respiratory health, these viruses also aggravate diseases including chronic obstructive pulmonary disease and asthma (Traves and Proud, 2007). The recent introduction (RSV and SARS-CoV-2) or increasingly widespread use (influenza virus) of vaccines against these pathogens led respectively to 35 to 64% (RSV) (Du et al., 2025), 90 to 95% (SARS-CoV-2) (Sheikh et al., 2021), and 42% (influenza virus) (Yegorov et al., 2025) decreases in hospitalizations among immunized adults, illustrating the potency of vaccines. However, despite their instrumental role in limiting the mortality rate, these intramuscularly administered vaccines proved insufficient to halt the transmission of pandemic pathogens and to provide long-lasting protection. This highlights the necessity to develop new effective vaccination strategies, with a particular focus on the protection of mucosal surfaces that constitute both the entry route and a major site of replication for most human pathogens (Tobias et al., 2024). To this end, it is essential to understand the distinctive features of mucosal immunity.

## 2 Mucosal immunity requires bespoke vaccines

The mucosa is the first line of defense against infections, allowing immunological monitoring. It stretches across an area approximately 200 times greater than the skin (Brandtzaeg, 2009; Mowat and Agace, 2014). The mucosa consists of an epithelial structure embedded with mucosa-associated lymphoid tissues (MALTs) such as the tonsils in the upper respiratory tract (Talks et al., 2024).

**Figure 1:**
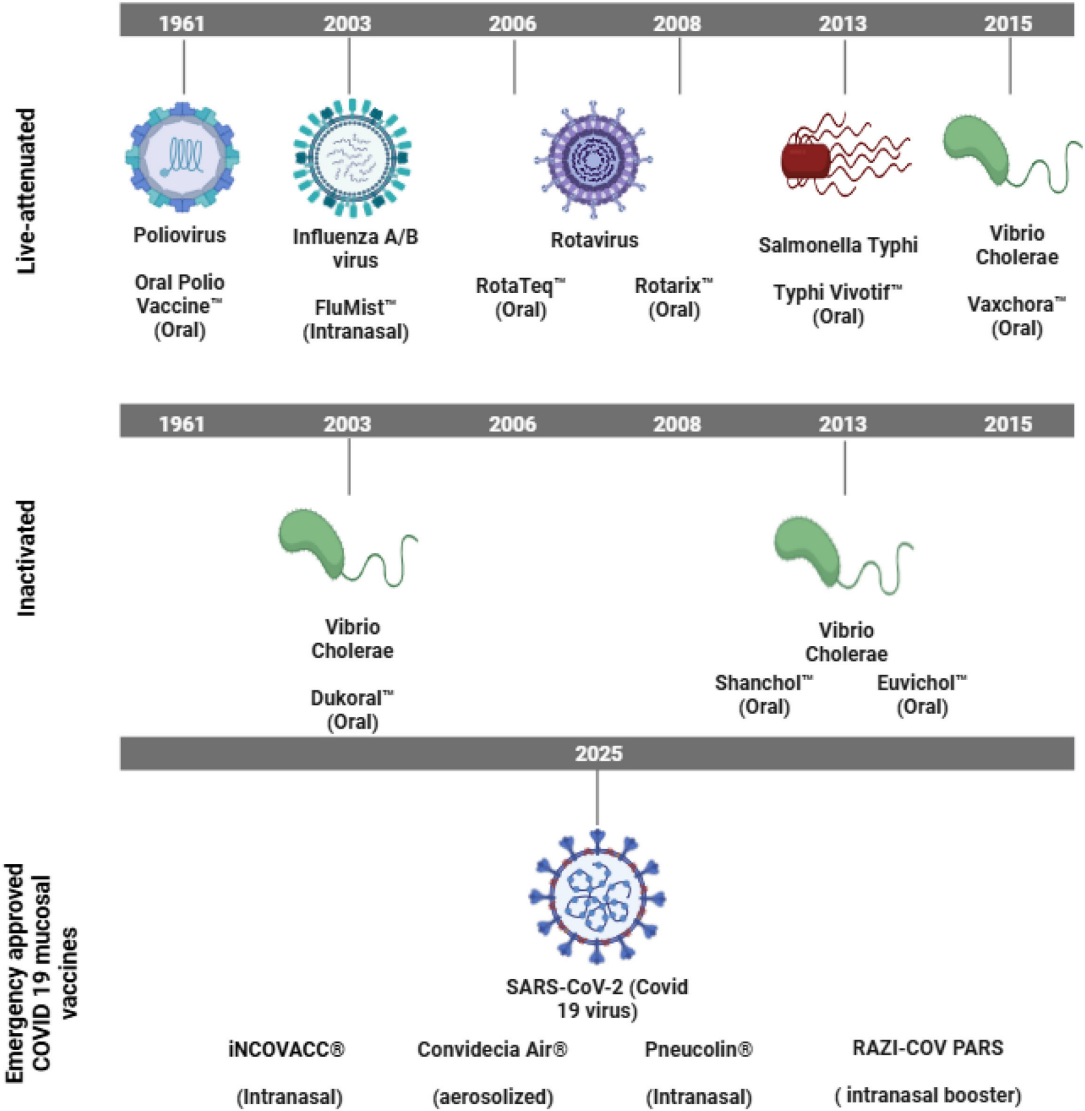
Clinically approved human mucosal vaccines. Overview of human-approved mucosal vaccines grouped based on the type: live-attenuated (top), inactivated (center), and nanoparticle-based (mRNA, adenovirus-vectored, virus-like particles; bottom). The target infectious agent, vaccination name, administration route, and year of approval are indicated. This figure was created using Biorender.com.

Mucosal immunity presents an important role in human defense by reducing the transmission of pathogens at their entry points (Miquel-Clopes et al., 2019; Tscherne and Krammer, 2025). Although promising, mucosal vaccines are rare, with only few of them having received human approval (**Figure 1**) (Shakya et al., 2016; Fraser et al., 2023). A number of persisting problems such as the enzymatic and structural barriers that hinder stability and antigen absorption, pre-existing immune tolerance, and the limited availability of potent licensed mucosal adjuvants (Rhee et al., 2012) hampers their widespread use. However, significant advantages such as the localized protection at infection sites and needle-free delivery (Song et al., 2024; Boyaka, 2017; Pilapitiya et al., 2023) might improve vaccine acceptance and coverage. Certain studies have also suggested that these vaccines are more effective when compared to systemic vaccines in preventing respiratory infections (Paris et al., 2021; Matsuda et al., 2021; Li et al., 2025), which has led to a spark of interest in mucosal vaccines, especially in the context of COVID-19 and influenza virus (Singh et al., 2023; Kastenschmidt et al., 2023).

Recent advances in regulatory frameworks, manufacturing methods, and delivery platforms designed specifically for mucosal immunization such as the inhalable or oral tablet forms are accelerating progress in this field (Zafar et al., 2023; Zhang et al., 2025).aApproximately 34 mucosal vaccine candidates reached clinical trials to date (Knisely et al., 2023; AbsolutelyMaybe.plos.org) indicating an emerging trend in diversifying mucosal vaccines despite the limited number of approved candidates.

One of the factors hampering the translation to the clinic of innovative mucosal vaccines is that animal models are poorly predictive of their immunogenicity in humans (Kraan et al., 2014; Jameson and Masopust, 2018; Kayesh et al., 2021; Gibbons and Spencer, 2011; Tscherne et al., 2025). This limitation has been attributed to interspecies variations in mucosal structure, innate immunity, IgA class switching patterns, and receptor expression. For example, rodents lack tonsils, which are a major site of mucosal immunization in humans, and contrary to human mucosal and lymphoid tissues cannot be infected by CD46-tropic adenovirus vaccine vectors (Sakurai et al., 2006). Consequently, unlocking the potential of mucosal vaccines requires experimental models that are more representative of human mucosal immunity may unlock the potential of mucosal vaccines (Kessie and Rudel, 2021), which is an area where human-derived *ex vivo* cultures are rapidly gaining relevance.

## 3 Emerging importance of *ex vivo* models

**Figure 2:**
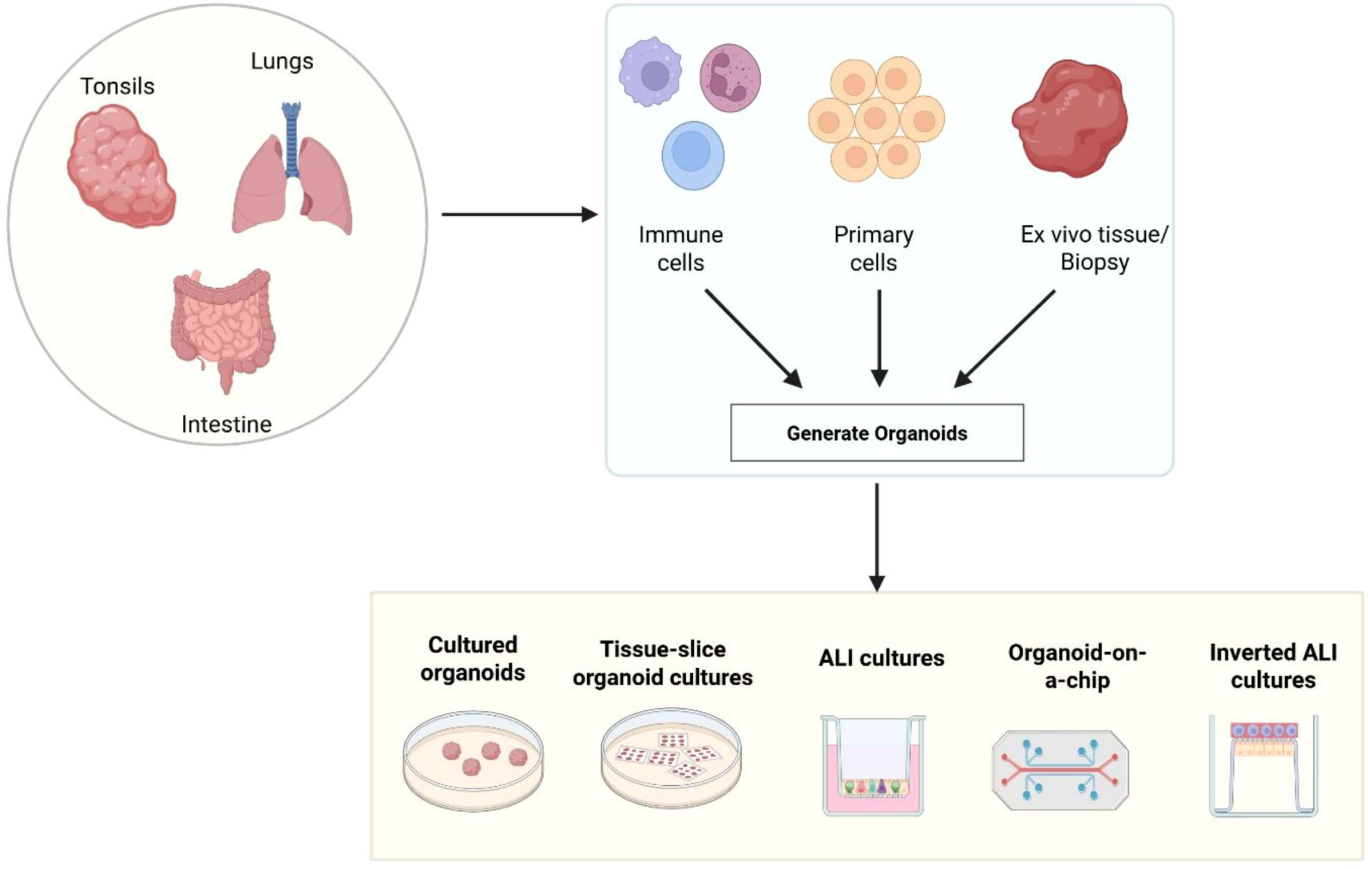
Overview of organoid generation from mucosal tissues and related culture methods. Organoids can be cultured from mucosal tissues such as lungs, tonsils, and intestines by utilizing the immune cells, primary cells, or ex vivo tissue biopsies. These organoids can be grown using various platforms, including standard organoid culture systems, tissue-slice organoid cultures, air-liquid interface (ALI) cultures, organ-on-a-chip systems, and inverted ALI cultures. These platforms are beneficial for exploring host-microbe interactions, mucosal biology, and immune responses in clinically relevant environments.

*Ex vivo* models derived from human primary tissues are recognized as critical tools to bridge the gap between *in vitro* and animal models. Building upon early 2D primary cultures or multicellular spheroids, the field of *ex vivo* models diversified exponentially in the 21^st^ century. Prominent systems include tumoral self-organizing organoids used for precision oncology and drug screening (Davies, 2018), and organs-on-chips relying on microfluidics systems (Ingber, 2022). Contrary to simple 2D cultures of immortalized cell lines, these platforms offer a more biologically relevant context to investigate immune activation, host-pathogen interactions etc (Fergusson et al., 2025). Unlike animal models, primary *ex vivo* models originate from human donors, and thus retain crucial immune cell composition, stromal components, and other species-specific characteristics (**Figure 2**) (Hoffmann et al., 1995). Here, we will present an overview of current *ex vivo* mucosal models and discuss how they have been applied or could be applied in the future to address critical challenges in mucosal vaccine development.

## 4 Current human *ex vivo* platforms for mucosal vaccine studies

Experimental models should be predictive of mucosal vaccines’ immunogenicity in humans. Consequently, highly valued readouts from ex vivo models include physiologically relevant antigen penetration and presentation, but also predictiveness of adaptive immune response features such as the clonality and neutralizing titers of humoral responses. In particular, primary adaptive responses have been more challenging to reproduce experimentally due to difficulties in maintaining physiological function of diverse interacting cell types and in reaching sufficient numbers of antigen-specific naïve lymphocytes. Models able to reliably mount such responses could be instrumental in the development of potent single-dose vaccines, a major goal of pandemic preparedness.

### 4.1 Two-dimensional cultures

The study of antigen penetration or pathogen infection, which is an integral part of vaccine development, requires organotypic (i.e., structural and functionally resembling native tissue) mucosal surfaces preserving physiological epithelial integrity and, in certain models, dynamic immune-epithelial interactions (Hewitt and Lloyd, 2021). To address this, cultures derived from primary human nasal epithelial or bronchial cells, such as MucilAir or Lonza’s NHBE airway models (Rayner et al., 2019; Aydin et al., 2021a, 2020, 2021b), have been developed. These models use the ‘Air-liquid interface’ (ALI) model, in which the apical surface is air exposed, while the basal part remains in contact with the culture medium. Respiratory ALI culture models may imitate the cellular complexity, differentiation, and polarization of respiratory epithelium, including ciliated and mucus-secreting cells, with intact tight junctions (Schogler et al., 2017). Such cultures support apical infection by respiratory viruses and enable for example the quantification of secreted mucosal cytokines, such as IP-10 and IL-6, in response to influenza virus (Perry et al., 2025).

In addition to conventional ALI platforms, inverted ALI cultures present a valuable method to investigate mucosal tissues *ex vivo*. In these systems, ALI cultures are temporarily cultivated upside-down in order to seed non-epithelial cells on the basolateral side of the porous membrane supporting the epithelial layer (Becker et al., 2024). For example, macrophage-epithelium co-cultures reacted to bacterial stimuli with cytokine secretion and macrophage cellular extensions (Noel et al., 2017). In addition, immortalized B cells facilitated the differentiation of part of the co-cultivated epithelial cells into microfold cells (also known as ‘M cells’), which are essential for antigen uptake at intestinal surfaces (Kimura et al., 2019; Wan et al., 2025). However, the Raji cells used in this platform lack certain functional characteristics of healthy B cells, so that obtained results should be considered with caution (Takakuwa et al., 2004).

Precision-cut tissue slices can be also cultivated from primary animal organs or human biopsies and preserve the *in vivo* tissue architecture. Though they have been used in numerous drug screening or pathomechanistic studies (Kuhl et al., 2023), their short lifetime in culture (Preuss et al., 2022; Fleischmann et al., 2025) hampers a broader use. Moreover, vaccine components introduced in the culture can reach the slice without crossing epithelial or other structural barriers normally present in tissues, thus limiting the use of tissue slices in vaccine development.

### 4.2 Explants and organoids

Full tissue explants can provide complex and organotypic co-culture systems of all cell types present in the mucosa of origin. Explants can be maintained in culture for short periods, or up to several weeks if microfluidic systems are used for steady-state perfusion of fresh nutrients and removal of waste products. Explants offer the advantages of low workload requirement and preservation of *in vivo* tissue architecture. Tonsil explants showed applicability as a vaccine screening platform, even showing primary adaptive humoral responses to the KLH protein (Bonaiti et al., 2024), which is a very potent immunogen. Explants can also be used to seed organoids to increase the culture lifetime, the diversity of structures that can be cultivated, and the experimental throughput due to organoid growth and dividing. For example, starting from lung explants, Choi et al. obtained immunocompetent organoids with an epithelial surface and a center composed of hematopoietic and stromal cells, that generated specific cellular responses to secondary infection (Choi et al., 2023). Similar intestinal models are frequently used in studies of inflammation, auto-immune diseases and colorectal cancer, though applications in vaccine study are still very rare (reviewed in (Kromann et al., 2024)).

Organoids cultivated from explants, induced stem cells or isolated primary cells have become effective *ex vivo* models that can demonstrate key aspects of secondary immune responses, including germinal center formation, antigen-specific B cell activation, and class-switch recombination (Wagar et al., 2021; Wagoner et al., 2024). For instance, lymphoid organoids obtained from human surgical tonsil samples represent a promising model to study mucosal immunology. Indeed, they contain abundant and functionally diverse populations, notably of T follicular helper cells (Tfh) and regulatory T (Treg) cells, which facilitate the formation of germinal center-like niches and the induction of adaptive responses in high density culture (Sureshchandra et al., 2024). Although different *ex vivo* models of lymph nodes have been described, tonsil lymphoid organoids have become highly prominent in the years 2021 to 2025 owing to their advantageous availability and ease of culture (Pudjohartono et al., 2025). Primary adaptive immune responses have been reported in these organoids (Wagar et al., 2021; Suhito et al., 2025), which shows the advantages of high-density culture to reconstitute physiologically-relevant immune activation pathways.

### 4.3 Organ-on-chips

Although traditional organoid cultures mimic key aspects of tissue architecture and function, they lack specific physiological cues such as vascular interactions and mechanical stress. To tackle this, organoid-on-chip platforms have been developed, further improving the physiological conditions of *ex vivo* systems (Papamichail et al., 2025). These models combine organoid cultures with microfluidics to mimic tissue *in vivo* conditions. For instance, tonsil organoid-on-chip models have been engineered to extend the lifetime of tonsil organoids and thereby facilitate a deeper, more physiologically representative study of adaptive immune responses (Teufel et al., 2025). Likewise, systemic lymphoid organ-on-chips obtained by high-density peripheral blood mononuclear cell (PBMC) cultures have been able to model differences in immunogenicity between mRNA COVID vaccines (Jeger-Madiot et al., 2024), suggesting that mucosal models cultivated with similar methods could be equally predictive.

On the other hand, lung-on-chip systems mimic the alveolar-capillary interface, enabling studies of fibrosis, viral infections, and drug responses (Bai et al., 2022). Similarly, other models include an ALI grown on top of a gel compartment containing stromal cells or macrophages (Lagowala et al., 2024). Apart from barrier and immune functions, organ-on-chips have also been employed to replicate microbial and mechanical factors in intestinal models that capture epithelial differentiation, peristalsis, and interactions with the microbiome (Ballerini et al., 2025). This highlights the significance of organoid-on-chip technologies to bridge the gap between traditional cultures and the complexity of living tissues.

Zhai et al. established a two-compartment system in which an mRNA vaccine was administered to a co-culture of myoblasts and antigen presenting cells (APCs). APCs were then transferred to a lymphoid follicle chip containing PBMCs for long term culture and stimulation of adaptive immune responses (Zhai et al., 2025). This model of systemic intramuscular vaccination was able to generate primary adaptive immune responses to rabies virus glycoprotein. This raises hopes that similar mucosal models could be developed using epithelial cultures instead of myoblasts and mucosa-associated lymphoid tissues instead of PBMCs to obtain all-in-one systems of mucosal vaccination.

## 5 Challenges of *ex vivo* human primary cell cultures

Although these successes highlight the multiple advantages of human *ex vivo* primary cultures compared to traditional animal models or established cell lines, certain technical and biological challenges have not been fully addressed to date. A number of experimental systems are still limited to a low throughput by low tissue availability, ethical requirements or technical complexity. However, certain models including tonsil lymphoid organoids or commercial ALI models are more accessible and benefit from a higher experimental throughput, while the relentless progress seen in the last decade already helped democratizing organ-on-chips technologies.

### 5.1 Experimental validation of physiological relevance

Despite constant progress, microfluidic, and organoid systems do not perfectly model spatial organization, cellular interactions or signaling patterns found *in vivo*, and lack inter-organ cross-talk that is critical in certain aspects of immunization. As a result, validating the biological relevance of immune responses observed in *ex vivo* remains a significant hurdle (Fleischmann et al., 2025). Many models use hyperphysiological cytokine doses to boost immune activation (Scalzone et al., 2023). Although this method can enhance assay sensitivity, it may also result in artificially elevated immune responses.

### 5.2 Variability

Studies involving human samples are highly susceptible to variability due to several factors including smoking habits of donors, age, and sex. This feature mirrors the variability of human populations and was harnessed to map donor-specific immune compartmentalization patterns using tonsil-derived organoids (Poon et al., 2023), but it also decreases the statistical power of experiments, in particular drug or vaccine screens. Although stratifying donors according to demographic or clinical background can enhance reproducibility, doing so might raise ethical concerns, especially with pediatric samples (Casati et al., 2022). To address inter-individual variability in *ex vivo* studies, some studies pooled primary cells across donors to normalize the donor-specific differences, thereby enhancing consistency across repeated studies (Kannan et al., 2024).

Establishing culture conditions, such as transwell insert formats, media composition, extracellular matrix coatings e.g., with collagen IV, and membrane pore sizes, are equally important for reproducibility (Chua and Lim, 2023; Pijuan et al., 2019). Nonetheless, challenges persist, such as material limitations, sensor integration, fabrication complexity, bubble formation, and technical reproducibility issues, especially in newly developed microfluidic systems (Leung et al., 2022). In particular, attempts to establish long-term co-cultures are frequently hindered by lack of compatible culture media supporting physiological differentiation and function of different tissues (Kessie and Rudel, 2021).

Therefore, to guarantee comparability, standardized experimental formats should be implemented in translational studies. For example, FDA guidelines for preventive and therapeutic vaccines suggest using the same batch of samples for non-clinical and clinical studies to ensure comparability and maintain consistency (FDA-2000-D-0029).

### 5.3 Microbiota influence on immune responses

An additional, yet often underappreciated, source of biological variability arises from the host-associated microbiome. The microbiome has a significant impact on mucosal immune responses by influencing tolerance, antigen presentation, and regulatory T cell differentiation (Belkaid and Hand, 2014). Microbial composition and diversity shape baseline mucosal immune activity and can determine the magnitude of vaccine-induced mucosal IgA and IgG as well as local T-cell responses (Ardura-Garcia et al., 2024). Microbiota-derived metabolites, like the short-chain fatty acids, can enhance epithelial barrier integrity and stimulate B cell class switch and differentiation into plasma cells, with likely implications on responses to vaccines. Furthermore, bacteria can play an adjuvant role, for example through TLR5 stimulation by flagellin (Lynn et al., 2022), one of the most potent mucosal adjuvants known to date (Rhee et al., 2012). Combined, these results indicate that targeting the microbiome, for example using probiotics and prebiotics, could enhance the immunogenicity of mucosal vaccines (Nieto et al., 2021). *Ex vivo* models incorporating microbial populations have already been developed and provided insights on colorectal cancer pathogenesis, tissue inflammation, and other phenomena (Puschhof et al., 2021). Future progress in co-culture systems may permit reliable studies of adaptive immune response in presence of bacteria and a broad usage of microbiota co-culture in vaccine development studies to model mucosal environments more accurately.

## 6 Future perspectives

Most mucosal COVID vaccines that reached clinical trials were repurposed systemic vaccines with little to no prior experience in mucosal delivery. For example, the ChAdOx1 and SAd36 adenovirus platforms were initially developed and tested for intramuscular vaccination (Antrobus et al., 2014; Fonseca et al., 2017). The former proved insufficiently immunogenic as a mucosal vaccine (Madhavan et al., 2022), while the latter was licensed as the iNCOVACC vaccine. However, it can be expected that the success rate of clinical translation would have been higher if suitable mucosal *ex vivo* models had been available instead of or complementarily with animal experiments. Likewise, the Flumist intranasal live influenza vaccine was reported to insufficiently protect against H1N1 viruses, because the H1N1 strain present in this multivalent vaccine tends to be outcompeted by other strains *in vivo* (Dibben et al., 2021). We thus hypothesize that using airway organoids during vaccine development may have helped to identify and address this limitation earlier.

The technical progress in *ex vivo* models align with regulatory innovations aimed at reducing the reliance of biomedical research on animal experiments, following the 3R principles. Notably, the recent FDA Modernization Act 2.0 authorizes clinical trials to be undertaken on the basis of *ex vivo* studies. This further increases the scientific and medical relevance of advanced culture systems. Nevertheless, mucosal vaccines still face regulatory hurdles as compared with the better-known systemic vaccines, in particular due to the scarcity of robustly validated mucosal correlates of protection (King et al., 2024). However, certain high-throughput *ex vivo* models may facilitate systematic screening of vaccinees’ serum or mucosal secretions for protection from pathogen infection in challenge studies. These experiments could help identify biomarkers of vaccination success based on multi-OMICs phenotyping of vaccinees’ samples (Kessie and Rudel, 2021).

Though the use of complex organotypic and/or immunocompetent cultures is still limited by technical skills requirements of lack of throughput or availability of tissues of origin, it is becoming increasingly accessible due to technical developments in culture longevity, readout sensitivity and more. For example, *ex vivo* models mounting primary immune responses, once considered unfeasible, are described on an increasingly frequent basis (Wagar et al., 2021; Zhai et al., 2025; Suhito et al., 2025)(We expect that increased cross-talk between scientific fields will help lower entry barrier and facilitate the adaptation of existing models, such as systemic lymphoid follicle-on-chips, to mucosal tissues. To fully realize the potential of mucosal *ex vivo* cultures, clinical validation of the immune response of each model would be required. Such experiments are still very rare both for mucosal and systemic cultures, going so far little further than recapitulating known immune activation mechanisms (Kastenschmidt et al., 2023) ex vivo, or showing that organoids grown from PBMCs of older donors secrete less antibodies than those of younger donors (Dauner et al., 2017). Therefore, ambitious studies comparing the immune response of *ex vivo* cultures to well-known vaccines with responses of tissue donors vaccinated after sample donation are warranted. Though not feasible for all models due to ethical requirements, these studies may prove critical in identifying best-in-class models and demonstrating clinical relevance. Together, these trends emphasize that while the field is advancing, bridging experimental progress with real-world impact will require coordinated innovation in immunology, engineering, and regulation.

## 7 Conclusion

The increasing burden of respiratory infections, worsened by concurrent outbreaks of SARS-CoV-2, influenza virus, and RSV, continues to represent a significant challenge on global public health systems, especially among vulnerable populations. While conventional systemic vaccines have considerably decreased hospitalizations and severity of the disease, their inadequate efficacy to prevent pathogen transmission highlights the need for novel mucosal vaccine platforms.

Mucosal vaccines provide localized immunity at the site of infection and therefore hold significant potential for infection control despite persisting challenges. However, biological, technical, and regulatory barriers such as mucosal tolerance, restricted antigen absorption, and the lack of approved mucosal adjuvants hinder their advancement (Sallard and Aydin, 2024). To overcome these, advances in vaccine administration methods such as licensed intranasal sprays and oral tablets or experimental microneedle patches (Paris et al., 2021) are paving the way toward the next generation of vaccines. Nevertheless, the inability of existing animal models and *in vitro* systems to precisely mimic human mucosal responses remain a challenge (Kayesh et al., 2021). Under these physiological constraints, *ex vivo* primary human tissue models, such as tonsil organoids or airway ALI cultures offer a more biologically relevant platform to investigate antigen penetration of target tissue or epithelial–immune interactions. Current models may be further refined to provide a holistic and accurate overview of the mucosal immunogenicity of candidate vaccines.

Furthermore, advanced immunophenotyping methods and standardized donor stratification might improve interpretability and reproducibility of existing models. Moreover, considering the significant influence of microbiome on mucosal immune regulation, including them into *ex vivo* cultures might also be beneficial.

In conclusion, mucosal vaccines are highly promising but remain an underdeveloped approach in respiratory disease control. To unlock their full potential, concurrent advances in tissue engineering, bioanalytical modelling and immunology are required. *Ex vivo* human-derived platforms may drive the design, evaluation, and translation of next-generation mucosal vaccines by merging experimental standardization and biological complexity, ultimately advancing effective vaccination strategies.
